# Is there any effect of pneumoperitoneum pressure on coagulation and fibrinolysis during laparoscopic cholecystectomy?

**DOI:** 10.7717/peerj.2375

**Published:** 2016-09-08

**Authors:** Turgut Donmez, Sinan Uzman, Dogan Yildirim, Adnan Hut, Huseyin Imam Avaroglu, Duygu Ayfer Erdem, Erdinc Cekic, Fazilet Erozgen

**Affiliations:** 1 Department of General Surgery, Lutfiye Nuri Burat State Hospital, Istanbul, Turkey; 2 Department of Anesthesiology and Reanimation, Haseki Training and Research Hospital, Istanbul, Turkey; 3 Department of General Surgery, Haseki Training and Research Hospital, Istanbul, Turkey; 4 Department of Anesthesiology and Reanimation, Lütfiye Nuri Burat State Hospital, Istanbul, Turkey; 5 Department of Ear Nose Throat Surgery, Lütfiye Nuri Burat State Hospital, Istanbul, Turkey

**Keywords:** Laparoscopic cholecystectomy, Pneumoperitoneum, Fibrinogen, D-dimer, Prothrombin time, Activated partial thromboplastin time, Thrombin time, International normalized ratio, General anesthesia

## Abstract

**Background:**

Laparoscopic cholecystectomies (LC) are generally performed in a 12 mmHg-pressured pneumoperitoneum in a slight sitting position. Considerable thromboembolism risk arises in this operation due to pneumoperitoneum, operation position and risk factors of patients. We aim to investigate the effect of pneumoperitoneum pressure on coagulation and fibrinolysis under general anesthesia.

**Material and Methods:**

Fifty American Society of Anesthesiologist (ASA) I–III patients who underwent elective LC without thromboprophlaxis were enrolled in this prospective study. The patients were randomly divided into two groups according to the pneumoperitoneum pressure during LC: the 10 mmHg group (*n* = 25) and the 14 mmHg group. Prothrombin time (PT), thrombin time (TT), International Normalized Ratio (INR), activated partial thromboplastin time (aPTT) and blood levels of d-dimer and fibrinogen were measured preoperatively (pre), one hour (post1) and 24 h (post24) after the surgery. Moreover, alanine amino transferase, aspartate amino transferase and lactate dehydrogenase were measured before and after the surgery. These parameters were compared between and within the groups.

**Results:**

PT, TT, aPTT, INR, and D-dimer and fibrinogen levels significantly increased after the surgery in both of the groups. D-dimer level was significantly higher in 14-mmHg group at post24.

**Conclusion:**

Both the 10-mmHg and 14-mmHg pressure of pneumoperitoneum may lead to affect coagulation tests and fibrinogen and D-dimer levels without any occurrence of deep vein thrombosis, but 14-mmHg pressure of pneumoperitoneum has a greater effect on D-dimer. However, lower pneumoperitoneum pressure may be useful for the prevention of deep vein thrombosis.

## Introduction

After the introduction of laparoscopic surgery, laparoscopic cholecystectomies (LCs) have been accepted as a gold-standard procedure for the symptomatic gall bladder stone (Himal, 2002; Suter & Meyer, 2001). In order to achieve better visibility of the surgical field, the “CO2 pneumoperitoneum technique” is used. There are many advantages to LCs such as a shorter hospitalization time, minimal postoperative pain and an easy recovery. However, there are also a few systemic disadvantages due to increases in intra-abdominal pressure. Insufflation of CO2 into the abdominal cavity results in elevation of the diaphragm and the risk of regurgitation, a decrease in lung volume and compliance, increment in airway resistance and an increase in the ventilation perfusion ratio. In the cardiovascular system, intra-abdominal pressure causes an increase in systemic venous resistance (SVR) and mean arterial pressure (MAP) and a decrease in venous return and cardiac output due to pressure on the inferior vena cava. If intraoperative CO2 pneumoperitoneum lasts a long time, renal artery flow decreases and results in a decreased glomerular filtration ratio (GFR) and urinary output. Mesenteric artery, intestinal mucosa, hepatic and splanchnic field perfusion decreases due to increases in intra-abdominal pressure (Diebel et al., 1992; Hashikura et al., 1994).

There are three major risk factors for deep vein thrombosis (DVT) during LCs: surgical trauma, pressure on the inferior vena cava and venous stasis on the lower extremities due to the anti-Trendelenburg position. Diagnosing DVT is difficult, but colored Doppler ultrasonography is a non-invasive and effective method for diagnosing DVT (Prone, Bounameaux & Perrier, 2001).

There have been studies advocating both mechanical and pharmacological DVT prophylaxis (London: Royal College of Physicians, 2010; Okuda et al., 2002), but there have also been other studies suggesting that prophylaxis during LCs is unnecessary (Agnelli, 2004; Blake, Toker & Dunn, 2001; Lindberg, Bergqvist & Rasmussen, 1997).

Some investigations have revealed increases in D-dimer and plasma fibrinogen levels during LCs (Milic et al., 2007; Vecchio et al., 2003). Coagulation and fibrinolysis cascade elements such as the prothrombin time (PT), the International Normalized Ratio (INR) and the activated partial thromboplastin time (aPTT) are some of the parameters that have been previously studied (Vecchio et al., 2003; Martinez-Ramos et al., 1999).

In this study, we aimed to record the coagulation factors and fibrinolysis response during different pneumoperitoneum-pressure LCs and to determine whether pressure decreases help to lower the risk of DVT.

## Material and Methods

### Study sample

After the Haseki Training and Research Hospital Ethics Committee (Location) approved the study (number 236) on 22 July 2015, 50 patients with symptomatic gall bladder stone who gave consent to participate and accepted the study’s terms were included. We focused on patients recruited between 24 July 2015 and 24 January 2016. All of the patients were diagnosed using hepatobiliary ultrasonography. Lower-extremity venous Doppler ultrasonography was performed on all patients the day prior to surgery and on the 7th postoperative day. Patients with DVT or a history of pulmonary emboli, anticoagulant usage, malignancy, acute cholecystitis, bleeding diathesis or individuals who were pregnant were excluded from the study. All of the operations were performed by same surgeon and anesthesiologist. All of the patients were diagnosed using ultrasonography by the same radiologist, and all cases of lower-extremity doppler ultrasonography were performed by a different radiologist.

### Surgery and anesthesia procedure

All surgical operations were performed successfully under general anesthesia without any complications. A standardized LC and anesthesia protocol were used for all patients. Propofol 2–2.5 mg/kg and fentanyl 1 µg/kg were used for the induction of anesthesia. Muscle relaxation for endotracheal intubation was obtained with rocuronium 0.6 mg/kg. Patients were ventilated with controlled ventilation (VCV) mode using an anesthesia device (Dräger Primus^®^; Dräger Medical Systems, Inc. Danvers, MA, USA). Tidal volume (Vt) was set as 6–8 ml per kg of ideal body weight and positive end expiratory pressure (PEEP) was set as 5 cmH_2_O. Respiratory rate was adjusted to maintain normocarbia (P_ET_CO_2_ = 32–36 mmHg). Maintenance of anesthesia was provided with sevoflurane (1.5–2%) with an oxygen-air mixture (FiO2 = 0.4). After the termination of anesthesia procedure patients were placed in Trendelenburg position. A small infraumbilical incisions were performed and a Veress needle was inserted. Pneumoperitoneum was achieved using CO2 insufflation. Patients were randomly assigned to one of two groups according to the pressure of pneumoperitoneum: the 10-mmHg group or the 14-mmHg group. Later, the patient was placed in reverse Trendelenburg position with a slight left angle. All of the patients were operated on using a standart 4-port LC technique. None of the patients received prophylactic Low Molecular Weight Heparin (LMWH). Elastic socks were used on each patient prior to surgery to prevent deep venous thrombosis. The patients were discharged between the postoperative 26th and 48th hours.

### Patient evaluation

All of the patients were examined in a detailed manner. Preanesthetic evaluation was performed by the same anesthesiologist the day before the of surgery. Age, sex, body mass index (BMI), American Society of Anesthesiologist (ASA) classification and comorbidities of the patients were recorded. Drain usage, surgical complications, surgery time, pneumoperitoneum time and oral intake time were also recorded.

Coagulation and fibrinolysis were assessed by prothrombin time (PT), activated partial thromboplastin time (aPTT), thrombin time (TT), international normalized ratio (INR) and fibrinogen and D-dimer levels from the venous blood samples obtained at the morning of surgery and one hour and 24 h after the surgery. An automatic coagulation analyser (Diagnostica Stago, STA Compact, France) was used for the analysis of blood samples. We used an STA—Neoplastine CI plus kit for the PT and INR measurements (normal levels 12–15 sec and 0.9–1.2), and we used the STA e PTT A 5 kit for the aPTT measurements (normal levels 28–40 sec). We used the STA—fibrinogen 5 kit for the fibrinogen measurements (normal levels 200–400 mg/dL). The STA Liatest D-DI kit was used for the measurements (normal <0.5 mg/L).

Serum alanine amino transferase (ALT), aspartate amino trasferase (AST) and lactate dehydrogenase (LDH) were measured before the surgery and 24h after the ssurgery to evaluate liver function. ALT, AST and LDH were measured using an automated AU680 clinical chemistry system analyser (Beckman Coulter Inc, Brea, CA, USA).

### Statistical analysis

We performed the statistical analysis using the SPSS software package for Windows (Statistical Package for Social Sciences, version 15.0; SPSS Inc., Chicago, Illinois, USA). Quantitative variables (age, weight, height, BMI, surgery time, pneumoperitoneum time and oral intake time, PT, aPTT, TT, INR, fibrinogen, D-dimer, ALT, AST and LDH) were expressed as mean ± standard deviation (SD) and/or median (min–max). Categorical variables (sex, ASA classification, coexisting disease, drain usage and surgical complication) were expressed as patient numbers. We analyzed the normality of quantitative variables using the Kolmogorov–Smirnov test, and we compared normally distributed variables using the Student’s *t*-test or to the Mann–Whitney U test when the variables were not normally distributed. The changes in PT, aPTT, TT, INR, fibrinogen, D-dimer, ALT, AST and LDH by time was investigated using repeated measures analysis of variance. Greenhouse-Geisser correction was used in the absence of sphericity assumption. Bonferroni test was used in postHoc multiple comparisons. Chi-square and Fisher’s Exact tests were used to compare the categorical variables between the groups. Based on a previous study D-dimer level after the LC with the 12 mmHg pressure of pneumoperitoneum was 315.26 ± 83.86 ng/ml. Power analysis with *α* = 0.05 and *β* = 0.2 for determining the 15% reduction on the D-dimer level with 10 mmHg pressure of pneumoperitoneum revealed that each group required a minimum of 24 patients (Garg et al., 2009). We adopted a value of *p* < 0.05 as being statistically significant.

## Results

**Figure 1:**
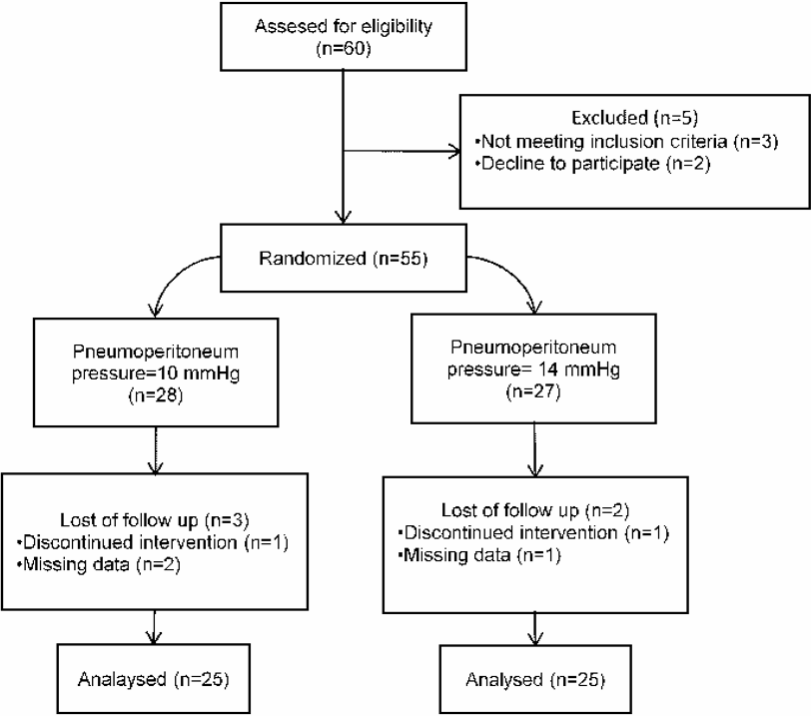
Flowchart diagram of the study.

Sixty patients who underwent LC due to symptomatic gall bladder stone were included this prospective randomized study. Ten patients excluded from the study. Fifty patients were randomly assigned to undergo LC with 10-mmHg of pneumoperitoneum pressure (*n* = 25), or 14-mmHg of pnumoperitoneum pressure (*n* = 25) (Fig. 1). Randomization was performed by a computer. The surgical procedure was completed successfully in all patients. Adequate exposure of surgical field was achieved in both groups of patients. There was no significant difference between the groups in terms of demographic characteristics, ASA classification, coexisting disease (hypertension, diabetes mellitus and chronic obstructive pulmonary disease), drain usage, surgical complication, surgery time, pneumoperitoneum time and oral intake time (Table 1).

We observed 1 postoperative wound infection (4%) in group I, which was easily managed with medical treatment. We used a negative-pressure hemovac drain for five patients (20%) in group I and three patients (12%) in group II due to minimal leakage from the liver bed. All of the drains were discharged on the 1st postoperative day.

Periphearal oxygen saturation was ≥98% and P_ET_CO_2_ was maintained between 32–36 mmHg by adjusting the respriratory rate. We didn’t observe hemodynamic adverse events during pneumoperitoneum such as hypertension (systolic arterial pressure ≥ 160 mmHg or diastolic arterial pressure ≥ 90 mmHg), hypotension (mean arterial pressure ≤ 70 mmHg), bradycardia (heart rate ≤ 50 bpm) or tachycardia (heart rate ≥ 100 bpm).

**Table 1:** Characteristics of the patients.

Characteristics	10 mmHg (*n* = 25)	14 mmHg (*n* = 25)	*P* Value
Age (y)	47 ± 15	52 ± 13	0.174
Gender, M/F	5/20	6/19	0.733
Weight (kg)	78 ± 13	76 ± 11	0.606
Height (cm)	165 ± 6	165 ± 6	0.930
BMI (kg/cm^2^)	28.1 ± 4.1	27.8 ± 4.5	0.819
ASA I/II/III	12/12/1	14/9/2	0.633
Co-existing disease			
Hypertension	6	8	0.529
Diabetes mellitus	4	5	1.000
COPD	2	2	1.000
Drain usage	5	3	0.702
Surgical complication	1	0	>0.999
Surgery time (min)	54 ± 9	57 ± 6	0.300
Pneumoperitoneum time (min)	35 ± 8	36 ± 7	0.393
Oral intake time (h)	8.44 ± 0.71	8.36 ± 0.70	0.772

**Notes.**

M male F female BMI body mass index ASA American Society of Anesthesiologist COPD Chronic obstructive pulmonary disease

We found a significant increase in PT 24 h after the surgery compared to preoperative value in the 10 mmHg group (*p* = 0.048) and the 14 mmHg group (*p* < 0.001). We observed a significant increase in APTT 24 h after the surgery compared to preoperative value in the 10 mmHg group (*p* < 0.001). In the 14 mmHg group there was a significant increase in APTT 1 h (*p* < 0.001) and 24 h (*p* < 0.001) after the surgery compared to preoperative value. There were significant decreases in TT 1 h and 24 h after the surgery in both of groups compared to preoperative values (*p* < 0.001). INR significantly increased in the 14 mmHg group 24 h after the surgery (*p* < 0.001). D-dimer and fibrinogen significantly increased in both of groups 1 h and 24 h after the surgery (*p* < 0.001).

Post 24 value of D-dimer was significantly higher in the 14 mmHg group compared to the 10 mmHg group. We didn’t observe any significant difference between the groups on pre, post1 and post24 values of PT, APTT, TT, INR and fibrinojen.

The PT, aPTT, TT, INR, fibrinogen and D-dimer values were summarized in Table 2.

**Table 2:** Comparison of coagulation and fibrinolysis between the groups.

	10 mmHg	14 mmHg	P1	P2	P3
PT					
Pre	11.94 ± 0.64	11.66 ± 0.79	–	–	0.175
Post1	12.10 ± 0.53	11.99 ± 0.57	0.345^a^	0.071^a^	0.505
Post24	12.43 ± 0.86	12.56 ± 0.66	0.048^b^	<0.001^b^	0.557
aPTT					
Pre	22.80 ± 1.48	22.01 ± 0.99	–	–	0.575
Post1	23.42 ± 1.54	23.32 ± 1.36	0.248^a^	<0.001^a^	0.129
Post24	24.54 ± 2.16	25.01 ± 1.65	<0.001^b^	<0.001^b^	0.429
TT					
Pre	19.88 ± 1.58	22,38 ± 4,78	–	–	0.055
Post1	17.78 ± 0.74	17.80 ± 2.68	<0.001^a^	<0.001^a^	0.971
Post24	16.06 ± 1.05	16.12 ± 0.95	<0.001^b^	<0.001^b^	0.833
INR					
Pre	1.00 ± 0.05	0.98 ± 0.06	–	–	0.183
Post1	1.02 ± 0.04	0.99 ± 0.04	0.199^a^	0.392^a^	0.058
Post24	1.04 ± 0.07	1.05 ± 0.05	0.067^b^	<0.001^b^	0.618
D-dimer					
Pre	0.31 ± 0.08	0.31 ± 0.07	–	–	0.306
Post1	0.56 ± 0.22	0.68 ± 0.24	<0.001^a^	<0.001^a^	0.307
Post24	0.91 ± 0.34	1.51 ± 0.30	<0.001^b^	<0.001^b^	<0.001
Fibrinogen					
Pre	224 ± 42	225 ± 28	–	–	0.906
Post1	278 ± 50	282 ± 42	<0.001^a^	<0.001^a^	0.797
Post24	356 ± 57	359 ± 42	<0.001^b^	<0.001^b^	0.802

**Notes.**

a Between pre-post1 values.

b Between pre-post24 values.

PT prothrombin time aPTT activated partial thromboplastin time TT Thrombin time INR international normalized ratio pre preoperative post1 postoperative 1st hour post24 postoperative 24th hour P1 within group (10 mmHg) P2 within group (14 mmHg) P3 between groups

There were no significant differences between the groups in terms of ALT, AST and LDH values. We found a significant increase in AST after the surgery compared to preoperative value (*p* = 0.015).

The ALT, AST and LDH values were summarized in Table 3.

**Table 3:** Comparison of ALT, AST and LDH values between the groups.

	10 mmHg	14 mmHg	P1	P2	P3
ALT (U/l)					
Pre	17 ± 10	22 ± 13			0.091
Post	19 ± 9	23 ± 8	0.438	0.767	0.066
AST (U/l)					
Pre	25 ± 10	21 ± 12			0.219
Post	26 ± 12	27 ± 10	0.769	0.015	0.703
LDH (U/l)					
Pre	208 ± 62	199 ± 61			0.603
Post	271 ± 200	232 ± 88	0.143	0.081	0.379

**Notes.**

ALT alanine amino transferase AST aspartate amino transferase LDH lactate dehydrogenase pre preoperative post postoperative P1 within group (10 mmHg) P2 within group (14 mmHg) P3 between groups

Normal serumvalues of ALT and AST were 0–35 U/l for female and 0–50 U/l for male. Normal serum value for LDH was 0–248 U/l.

## Discussion

Pneumoperitoneum applied during LCs may result in many changes in the cardiovascular, hormonal and neuroendocrine systems. There have been reports of sudden cardiac arrest or pulmonary edema cases in the literature. Additionally, decreases in venous return, portal vein flow and intra-abdominal organ perfusion have been associated with pneumoperitoneum pressure in previous studies (Joris et al., 1993; Jakimowicz, Stultiens & Smulders, 1998).

According to the International Abdominal Compartment Syndrome Consensus Conference 2004, intra-abdominal pressure is accepted as a steady-state pressure; it increases with inspiration, decreases with expiration and correlates with directly solid organ volumes, ascites, blood, lesions occurring (tumor, pregnancy) and lesions restricting the expansion of the abdominal wall (burns, scars, edema). Compression effects and the peritoneal absorption of CO2 increase cardiovascular and pulmonary complications (Malbrain, 2001; Windberger et al., 1999).

In the present study we didn’t find any significant difference between the groups with respect to the the ALT, AST and LDH values. In 14 mmHg group there was a statistically significant increase in AST value postoperatively, but this was not clinically important.

There may be a transient postoperative hepatic transaminase increase associated with laparoscopic surgeries. This increase is believed to be associated largely with CO2 pneumoperitoneum. In the majority of cases, this transient increase is resolved without any clinical findings. Tan et al. (2003) compared laparotomy and laparoscopic surgeries with postoperative serum liver enzymes. They observed a larger increase in AST and ALT levels during the postoperative 24–48th hours for laparoscopic cases than for laparotomy cases. There was a slight increase in total and direct bilirubin levels, but no changes were observed in ALP, LDH or GGT levels. Giraudo et al. (2001) reported that there were very few changes in hepatic parameters comparing 14 mmHg CO2 pneumoperitoneum and gas-free laparoscopy. According to Bendet et al. (1999), postoperative aminotransferase levels increased due to Kupffer and endothelial cell injury, especially during LCs. Volz et al. (1999) reported that increases in intra-abdominal pressure over a short period of time may affect portal vein flow. These authors demonstrated an ondulation in portal vein flow. They reported that this ondulation causes a reperfusion injury, particularly in the Kuppfer and endothelial cells lining the hepatic sinusoids. This injury in turn resulted in a liver enzyme increase. In this study, we found an increase in blood coagulation parameters during the postoperative period. This increase is believed to be related to the Kupffer and endothelial injury due to intra-abdominal pressure and hepatobiliary manipulation. Intra-abdominal pressure affects the cardiovascular system via an increase in systemic vascular resistance and mean arterial pressure and a decrease in venous return and cardiac output due to pressure on the inferior vena cava. If the intraoperative CO2 pneumoperitoneum lasts for a longer period of time, this may result in a decrease in renal artery flow, GFR and urinary output (Joris et al., 1991; Takrouri, 1999). The pressure effects of pneumoperitoneum can be partially prevented by applying a lower insufflation pressure (Neudecker et al., 2002; Grusamy, Samraj & Davidson, 2009). In our study, we compared the effects of 14 mmHg and 10 mmHg pressure pneumoperitoenum on coagulation parameters (i.e., aPTT and PT). We found a statistically significant increase in both of the groups. Furthermore, we found an increase of aPTT and PT levels in the higher-pressure group, but this difference was not statistically significant.

An LC carries a low risk of DVT (Blake, Toker & Dunn, 2001). Although INR, and fibrinogen increased in the 24th postoperative hour, affects and disturbs the coagulation cascade, there are studies that present contradictory findings (Marakis et al., 2006). It has been shown that D-dimer was the only true sensitive marker of coagulation and fibrinolysis whereas the other parameters such as PT, APTT are indirect indicators of coagulation of fibrinolysis (Milic et al., 2007). It has been reported on previous studies that pneumoperitoneum during LC may cause coagulation and platelet activation and resulted in postoperative hypercoagulabilty (Amin et al., 2014). Ido et al. (1995) has shown that blood flow velocity in the femoral vein was reduced during pneumoperitoneum proportionally with the magnitude of intraabdominal pressure. However, increased intraabdominal pressure leads the venous pooling in the lower extremities.

In the present study, significant increases were seen in the postoperative levels of INR, D-dimer and fibrinogen in both groups. The only difference between the groups was observed in D-dimer after the surgery. We found that higher pressure of pneumoperitoneum cause more increase in D-dimer after the surgery. A three- and five-fold increase in D-dimer in the 10 mmHg group and in the 14 mmHg group, respectively, were seen 24h after the surgery.

Garg et al. (2009) reported on 50 LC cases with standard 12 mmHg pneumoperitoneum pressure. These authors found that aPTT and antithrombin 3 levels decreased at the postoperative 6th and 24th hours and that these decreases resulted in activation. Furthermore, they did not observe any instances of clinical DVT in their patients. Another study by Ntourakis et al. (2011) reported that PT, aPTT, INR, D-dimer, fibrinogen and FDP levels increased during the postoperative period in a statistically significant way. We also noted a significant increase during the postoperative 1st and 24th hours in PT, aPTT, INR, fibrinogen and D-dimer levels. At the same time, the TT, which is associated with the intrinsic and extrinsic pathways of coagulation cascade, significantly decreased within the groups. However, when we compared the different pressure groups similar to other parameters, there was no significant difference in TT. According to the literature, this increase in PT and INR levels is believed to decrease the DVT risk associated with LCs (Morino, Giraudo & Festa, 1998; Hasukic, 2005).

We did not find any differences between the groups in terms of demographics, anesthesia technique, surgical technique or operation time. The only difference was the CO2 pneumoperitoneum pressure difference between the groups. We only recovered a statistically significant difference in D-dimer levels, although we noted an increase in PT, aPTT, INR and fibrinogen levels in the 14 mmHg pressure group. We additionally recovered a more pronounced decrease in TT levels in the 14 mmHg pressure group, although this finding was not statistically significant. Based on our results, we can conclude that higher-pressure (14 mmHg) pneumoperitoenum has a more negative effect on coagulation factors and fibrinolysis than lower-pressure (10 mmHg) pneumoperitoenum.

There have been other studies comparing laparoscopic and open cases that revealed similar results for coagulation factors (Martinez-Romos et al., 1998; Vander Velpen et al., 1994). However, there has been no study thus far comparing the effect of pneumoperitoneum pressure levels on coagulation cascade and fibrinolysis. In this investigation, we found more negative effects on the coagulation system with higher pressures.

## Conclusion

We found that pnemoperitoneum conducted using 10 mmHg and 14 mmHg pressures has a similar effect on human hemodynamics and metabolism and resulted in a significant increase in coagulation factors in both groups. According to increases in coagulation parameters, higher CO2 pressure (14 mmHg) pneumoperitoneum has a more negative effect on coagulation cascade and fibrinolysis. All these changes were reversible, and we did not observe any complications in either group. We did not encounter clinical or ultrasonographic DVT or pulmonary emboli in this study population. Based on the results of this study, we suggest a lower pressure of pneumoperitoneum pressure (10 mmHg) during LC because of the less pronounced effects on the coagulation and fibrinolytic system.

##  Supplemental Information

10.7717/peerj.2375/supp-1 Data S1 Raw data: documentation of patients including the study

10.7717/peerj.2375/supp-2 Data S2 Raw data: final corrected version of results of study

## Additional Information and Declarations

### Data Availability

The following information was supplied regarding data availability: The raw data has been supplied as Supplemental Files.
